# Hospital and Institutional News

**Published:** 1914-09-19

**Authors:** 


### HOSPITAL AND INSTITUTIONAL NEWS.

### "THE HOSPITAL'S" SPECIAL CORRESPONDENTS.

While thanking our special correspondents all over the country for their ready assistance and regular weekly reports, and regularity is of prime importance, there seems to be a slight misapprehension on the part of one or two on the subject of newspaper cuttings. The scissors and paste method, while showing good-will, does not constitute a correspondent's original matter, which, however, is precisely what is required, partly because hospital men naturally understand what points to bring out far more than any lay reporter can do, but chiefly because the news comes authoritatively and first-hand. That is the overwhelming reason why we must ask all our correspondents to be good enough to write their own reports (which the responsible positions of the large majority ensure their being able to do), and not to be content with sending a few cuttings from local newspapers. We need hardly add that the best and fullest accounts have always come to us in original form, and that there are only one or two who have been content merely to refer to a local newspaper.

### THE KING'S ATTITUDE TO GERMAN PRISONERS: A NATIONAL CONTRAST.

A magnificent example was set by the King on his visit, accompanied by the Queen and Princess Mary, to Netley Hospital, Southampton, on Tuesday, when he made special inquiries in the wards set aside for Germans as to the arrangements made for their comfort. He expressed his desire that everything should be done to make them as happy as possible. On the very day that this visit is recorded in our newspapers, the *Times* contained, among the items officially circulated through German wireless stations and received by the Marconi Company, news that German newspapers publish numerous letters from the field complaining bitterly of reports that German women and girls have received French and Russian prisoners with kindness. One German soldier's letter is quoted as follows: "We are all filled with disgust at such behaviour. Women like this ought to be put in front of the troops in order that they might feel in their own bodies what our lot is." Is it a coincidence that the country of which King George is the sovereign is the home and pillar of the voluntary system of hospital support, and that the Germany which repudiates kindness and charity to non-combatants, whether prisoners of war or women and children, is ridden by a State system of hospitals in which the rights of "science" rather than of the patients are given the first place? We think that there is matter for thought in this contrast.

### THE KING'S VISIT TO NETLEY.

At Netley Hospital, where the above remarkable incident took place, are some five hundred sick and wounded. The Royal party, who arrived before two o'clock, allowed a Red Cross train at the hospital station to proceed with its work of disembarking wounded Germans brought from Southampton Docks, and in consequence arrived at the hospital station after a delay of twenty minutes. At the institution the King was received by the officer-in-charge, Colonel D. J. R. Lucas, C.B., R.A.M.C., and other officials. The Royal party was conducted over all the wards, remaining a considerable time in those in which Germans were accommodated, and talking with them in German. One of the German officers, who could speak English, expressed on behalf of his fellow-prisoners their thanks for the King's visit and consideration.

### AN IRISH AMBULANCE TRAIN.

An enterprising step has been taken by the Great Southern and Western Railway Company in Ireland, which, to meet the possibility of wounded being landed on the south coast, have built and equipped at their Inchimore works a complete new ambulance train. By the adaptation of nine bogie coaches ordinarily used for main line parcel traffic, five "wards," with twenty beds each, have been fitted in two tiers. To each car an end gangway has been fitted, by which access is gained to the pharmacy car, which is given the central position. The so-called pharmacy car comprises in separate divisions a surgery, which is zinc-lined, pharmacy, linen stores, medical officer's room, and stores for bandages and other equipment. The corridors are made wide enough to accommodate a stretcher, while a sliding door is fixed so as to allow a patient to be taken sideways into the surgery. There is a dining-room car for the staff, fitted with lavatory accommodation, and the seats are upholstered in such a way as to be convertible for sleeping purposes if required. There is also a sleeping saloon for the medical and nursing staff, with beds for two doctors and two nurses, and, divided off, for two attendants. A stores car, more or less on the ordinary corridor plan, with a final guard's van, brings up the rear. The ventilation and lighting have been carefully studied, and electric bells and steam heating are provided throughout the train, which is nearly two hundred yards in length.

### THE WAGES OF PANEL PRACTICE.

Some very interesting returns, indicative of how much panel doctors receive for their services, are forthcoming from Sussex, and, as suggestive of the ratio of pay to work under the panel system, may be briefly summarised here. At a meeting of the West Sussex Insurance Committee last Saturday the following figures were made public. The total average number of insured persons entitled to medical benefit during last year was 49,559, and the average number on the doctors' lists was 43,612, or an average to each panel practitioner of 366. The largest number on any single doctor's list was 1,462, and the smallest, one. The amount which had been allocated, representing those insured persons who did not select a panel doctor was, in round figures, £1,146. For the year the total sum due to the doctors was £18,383, or an average per doctor of £154. But in the settlement for the year fifty-three doctors would receive less than £100; twenty between £100 and £200; twenty-four between £200 and £300; thirteen between £300 and £400; four between £400 and £500; one between £500 and £600; and one between £600 and £700. What does the National Medical Union make of these figures? We should be interested to hear the secretary's analysis of them. Do they not provide the non-panel men with a capital test case?

### HOSPITAL APPEALS IN TIME OF WAR.

What should be the attitude of voluntary hospital secretaries and treasurers to the public at the present time? is a question easier to ask than to answer. Yet it is one to which an answer must be found, since a large proportion of money is and will be undoubtedly diverted to the central and local relief funds. At a time of difficulty any suggestion may prove helpful, and, therefore, we quote one lately made by an institutional secretary in Glasgow, as much in the hope that it will provoke managers to send us others as for its intrinsic value. He urges on managers, treasurers, and secretaries that they would be much more unlikely to receive such answers as "No contribution this year" or "Not in the meantime" if they made a point of asking for small contributions, a half or even a quarter of that previously given, than formally for, in the stale phrase, "a renewal of your support." The suggestion is one of tactics and as such has its value, and, like all tactical suggestions, almost everything depends on the way in which the request is put. To ask for half a man's former subscription would be manifestly ridiculous, but to say "If on account of the war you feel tempted to curtail your expenditure by giving little or no help to charitable institutions, will you at least resolve not to send less than one-half of your previous subscription to us?" might prove fetching. The question really is whether tactics alone of this kind are sufficient to meet the difficulty. Is not a complete policy required? Is it not possible to use the war itself, seeing what the hospitals are doing for the wounded, as a lever to raise the wind? We shall be glad to publish any suggestions on the point. What are our readers' opinions?

### BRITISH COLONY'S HOSPITAL, ANTWERP.

The British colony in Antwerp has equipped a hospital in a building lent to them by the Missions to Seamen. This has, of course, already been busily occupied in the treatment of the wounded, and the *Times* correspondent reports a visit from the Queen of the Belgians to the institution on Monday. She was received by the British Minister, Sir Francis Villiers, and by two members of the committee, Mr. W. H. Newman and Mr. L. Evan Thomas. The medical and nursing staffs had the honour of being presented, and the Queen spent some time in examining the $x$-ray department, the operating-theatre, and the kitchen. Every wounded soldier received a handshake and a few words. The visit has caused great delight not only to the patients, staff, and indeed the whole British colony, but is welcomed by all as another expression of the common aims and bonds of fellowship among the allied nations.

### INSURANCE COMMITTEES AND SOLDIERS' DEPENDANTS.

We are glad to see beginnings of an awakening to the fact that prompt steps must be taken by the public authorities to see that the relief granted to dependants of insured persons who are engaged in the defence of their country must not have even a semblance of Poor-Law methods of distribution. An example comes to hand from Bristol which illustrates this welcome tendency. At a recent meeting of the Bristol Insurance Committee, over which Dr. Walter Saise presided, a motion was put down in his name calling attention to the decision come to at the Council House on August 12 that the relief and assistance would be given at the Poor-Law centres. Dr. Saise stated that as these payments were not Poor-Law relief, but a free gift from the public to the wives and children of those engaged in their defence, he had felt very much concerned at the plan adopted at that meeting. He had since learnt, however, from the Town Clerk that steps had been taken to remove the relief centres from the Poor-Law offices and to constitute the central office of inquiry at the Council House. He therefore withdrew his resolution and substituted one expresing the Insurance Committee's satisfaction that arrangements had been made under which it was no longer necessary for the dependants of insured persons on naval and military service to apply at Poor-Law centres when in search of assistance. The original and fortunately now discarded proposal seems to have been adopted because it provided machinery of a kind ready to hand which had been offered to and accepted by the Council.

### THE BESETTING DANGER OF RELIEF FUNDS.

Those who read this week's leading article will realise that this example of taking the line of least resistance, however natural at the beginning of an emergency, is just the way in which a disastrous procedure springs up. We are very glad that the error has now been rectified. As to relief in general, there has been a bad tendency up to date to devote more energy to the collection of money than to the collection of information as to where that money is required. Yet surely every possible assistance should be given to make those who need assistance find the path to relief an easy one. To put the matter in its extremest form, it is better that a few undeserving cases should receive money than that a few deserving ones should not. It cannot in any case be too much emphasised that the true difficulty is not to raise the necessary money, but to find out all those entitled to it.

Our duty does not end in raising the money for the dependants of those on active service. On us, and not on them, must lie the onus of arranging for applications for help. We must bring the gifts to them, and not thrust on them the hardship of applying to us.

### THE FIRST AUXILIARY RED CROSS HOSPITAL.

At the request of the military authorities, the first auxiliary Red Cross hospital in Scotland has been established at Haddington. It is designed to meet the requirements of the body of troops which has been stationed in the neighbourhood; in fact, the origin of the hospital arose out of the fact that certain troops were hurriedly despatched to the district without sufficient time being allowed for the provision of arrangements for the treatment of their sick. When they arrived the medical officer called upon the Voluntary Aid Detachment, through the local Territorial Association, to supply temporary hospital accommodation for those of the men who had been overcome by the effects of their forced march. Since the military authorities have felt the need for its continuance, they have asked the Scottish Branch of the British Red Cross Society to recognise it as a small auxiliary hospital in which slight cases requiring temporary treatment might be received. In the meantime the Red Cross authorities have visited the institution, and, finding everything to be satisfactory, have acceded to the request made by the military authorities.

### AN ENTHUSIASTIC WORKER IN CUMBERLAND.

Those interested in the working and efficiency of the Cumberland Infirmary will have been pleased to learn the recent election of Mr. E. T. Tyson, High Sheriff of Cumberland, to the committee of the institution in place of the late Mr. H. C. Howard. In a twofold manner is the appointment an interesting one. Mr. Tyson was one of the first supporters of the infirmary to endow a bed, thereby setting a valuable example; he also gave the first open-air shelter which was provided in connection with the wards. In the second place, he represents a district in West Cumberland from which a large number of patients come, and consequently provides an official link between this district and the hospital. Mr. Howard, whom Mr. Tyson succeeds, was a vice-president, and had been interested in the Cumberland Infirmary for many years.

### AN OXFORD COLLEGE CLOSED.

News has come to hand in several instances of the temporary conversion of colleges at our universities into hospitals, and all who remember Neville's Court at Trinity College, Cambridge, will realise how easily and well cloisters can be adapted as open-air wards, almost as if they were actual hospital balconies, except that two sides are deprived of sun. But, so far as we know, Ruskin College, Oxford, is the first there or at Cambridge to be closed for the 1914-15 session on account of the war, and now the college is to be offered for hospital purposes to the Government. It will be recollected that apart from resident students, for whom it will now not be open, Ruskin College also conducts a correspondence department for non-resident students, and this department will be carried on as usual. It would be interesting to know how far the effects of the war on the particular class of men for whom Ruskin College caters is responsible for the decision of its executive committee to close it. But in the meantime its offer for hospital purposes awaits acceptance, and, if acted upon, will leave it, we believe, the sole college in the older universities entirely "hospitalised" on account of the war.

### POOR-LAW INSTITUTIONS AND THE WAR.

The Brentford Union are offering accommodation for 250 patients in their various institutions to the War Office, Leicester parish a ward of thirty-two beds at the North Evington Infirmary, the Dewsbury Union fifty beds in their infirmary, the Kingston Union (Herefordshire) the use of the Workhouse Infirmary, the Sturminster Union (Dorsetshire) twelve beds, and the parish of Alverstoke (Hampshire) a block of the workhouse. In the Bristol Union 100 beds have been prepared for use in the new Southmead Infirmary, and cases are expected there in the course of a few days. These facts form a brief summary, and provide a bird's-eye view of the important share which the Poor-Law infirmaries are taking in providing accommodation for the sick and wounded on their return from service abroad. The arrangements for the reception of wounded at Southmead Infirmary are being pushed on rapidly, and it would now be possible to receive some 300 men if necessary.

### INFIRMARY BEDS FOR THE WOUNDED TROOPS.

The City of London Board of Guardians have adopted the suggestion made on August 15 in the columns of The Hospital of placing at the disposal of the War Office their vacant beds in the infirmary. At the last meeting the Homerton Committee drew attention to the growing need for hospital accommodation for sick and wounded troops daily returning from the seat of war. The committee reported that in the infirmary block at Homerton there were fifty-four beds which could be conveniently placed at the disposal of the War Office for this purpose without in any way interfering with the ordinary requirements of the establishment. Therefore they recommended that these beds should be offered to the authorities through the Red Cross Society. The recommendation was unanimously adopted by the Guardians.

### THE "SENIOR" RED CROSS ORGANISATION.

Appeals are not supposed to make very interesting reading, but few could fail to be struck by the organisation disclosed in the letter now issued under distinguished signatures on behalf of the English Order of the Hospital of St. John of Jerusalem, more commonly known as the St. John Ambulance Association. The writers point out that, dating back eight hundred years, it is "the senior Red Cross organisation in this country, and, in fact, the St. John Ambulance Association was nearly forty years ago the pioneer of all 'ambulance' work." This was in 1877. The work of the St. John Ambulance Brigade, which, strictly speaking, is an offshoot of the Order, is carried on mainly by the industrial classes, and makes little stir in the world; but if an answer be wanted to the question, "In all this Red Cross work, where do the orderlies come from?" the Brigade supplies the answer. No fewer than 5,000 men belonging to it have been mobilised and are serving as hospital orderlies with the Army and Navy. Some 130 trained nurses have also been sent out. The ambulance department succeeds in maintaining itself in time of peace, and the members of the Brigade largely raise their own funds. Its classes for instruction in first-aid, given by qualified medical men, have been a well-known feature, and the number of orderlies which it has been able to send out prove the value of the corps which it forms for the transport of the wounded, a work, it may be added, for which its practical and constant experience in the transport of injured in time of peace has provided the best possible training. As a centre for the regular training of hospital orderlies, whether in peace or war, the St. John Ambulance Association deserves exceptionally well of the public at the present time.

### RUBBER FLOORS FOR HOSPITALS.

We have previously alluded to the gift of rubber to Guy's Hospital, London, and to the Royal Infirmary, Edinburgh, whereby the Rubber Growers' Association hope to demonstrate the value of this new material for floors and, we are told, even for street purposes. Now that Mr. John M'Ewan, chairman of the Association, has recently handed over the material to the managers of the Edinburgh institution a few points concerning its extent and design become of interest, though a prolonged trial will, of course, be necessary in order to gauge its utility through practical experience. Some 800 square yards have been presented to the Royal Infirmary, which are used to cover the whole of the main right and left wing corridors and the entrance hall. The local firm known as the North British Rubber Co., Ltd., of Castle Mills, Edinburgh, have manufactured the rubber, which has been laid down in a black-and-white pattern from Mr. Frank Edward B. Blanc's designs. He has chosen for the corridors a black-and-white square design, suggestive of a tesselated pavement, but in the central hall, which is his most ambitious effort, on a white background surrounded by a classic border appears in black the arms, crest, and motto of the Royal Infirmary. In view of this experiment in flooring it is interesting to place on record at the time of laying the claims for rubber in this respect. The chief is its durability, which is claimed to be almost everlasting. In addition, and more obviously, the rubber floor is practically silent, pleasant and resilient to the tread, and, moreover, warm to the feet. The price is stated to be little more than that of marble, and almost any colour scheme is said to be capable of reproduction in it. Since probably the floor which exacts the severest hospital test is that of the theatre, its fitness or otherwise for this becomes the supreme question; for the theatre floor not only receives hard wear, but must possess in a high degree easy cleansibility and non-absorbable qualities, together with unaffectability by high temperature. How far in practice these qualities are found to be possessed by rubber it would be premature to decide. We hope to return to the subject shortly.

### THE PROBLEM OF LIGHTING EMERGENCY HOSPITALS.

When a building is hurriedly taken over for hospital purposes at the present time, in spite of the inspection to which it is subjected, there will be an inevitable tendency to pass as "good enough" many things which do not really come up to the hospital standard. The sometimes loudly expressed disappointment, almost verging on anger, when this or that marked-out school or hall has subsequently been refused by its owners or condemned for its structural or situational inconvenience by hospital or military authorities, is an obvious indication of this. One of the defects likely to be passed is the lighting. We may assume that the gas brackets or electric globes in all taken-over buildings will be left much as they were before preparations for the wounded were thought of. The *Lighting Journal*, therefore, is doing something useful in pointing out that what it calls the "light comfort" of the patients in these emergency buildings should be carefully studied. It alludes to the necessity of arranging for screening the rays from the beds, and urges that "improved lighting methods" should be fitted in all temporary establishments for the wounded. If the *Lighting Journal* were written valiantly up to its title it would follow this with a technical description of these improvements, for many kinds have been devised, but it leaves them injudiciously vague, and thereby robs much of its argument of proper value.

### TWO PRACTICAL LIGHTING SYSTEMS.

However, in The Hospital of May 3, 1913 (page 180), we devoted a special article to the subject of "The Artificial Lighting of Hospital Wards." In that article, which contained illustrations of the principle described, we referred to the new "eye-rest" fittings which had been installed at the Chiswick Cottage Hospital through the efforts of Messrs. Scott and Fraser, architects, in conjunction with the British Thomson-Houston Company. The lamps designed by the latter are contained, we noted, in bowls which are fitted with silvered-glass reflectors that direct the light from the filaments on the ceiling from which it is radiated downwards as a diffused light. This method is, of course, for electric lighting, and though this is common to-day, it cannot be expected universally in buildings merely adapted in a hurry for hospital purposes. Again, in the *Institutional Worker* Section of The Hospital on May 30, 1914, we published an account of "A Cheap, Efficient, Automatic Electric-Lighting

System," giving the experiences of a sanatorium with the Lister-Bruston system. Improvements also in gas have been devised, but we have said enough here to show that fittings exist which may easily transform the lighting of a lay building into one not unsuitable for a hospital ward. And if the firms which have specialised in this branch of illumination are awake to their opportunity, they will lose no time in letting the public know what their inventiveness has made available.

### THE MEDICAL TREATMENT OF REFUGEES.

The concentration of refugees, especially from Belgium, in large centres reminds us that our hospital problems do not come to an end with the provision of medical care and treatment for the sick and wounded. Private generosity and enterprise cannot obviously cope with the demand for accommodation of those numbers who have now sought refuge on our shores from the burnt or shattered cities of Belgium. Thus concentration camps, as they may conveniently, if not entirely accurately, be termed, are springing into existence. As the Government is providing the ships in which the refugees are brought to England, and is responsible for their subsistence and accommodation, the aid of public authorities is being invoked for dealing with them. Thus the Local Government Board is understood to be using the Alexandra Palace as a receiving centre, with the result that the sanitation and medical organisation of this large aggregation of men, women, and children becomes a pressing problem. So the apparently inexhaustible versatility, good-will, and organisation of the Metropolitan Asylums Board are being invoked, and Dr. Herbert E. Cuff, the Board's medical officer for general purposes, has been appointed medical superintendent. How large the task before him may be is to be judged from the fact that preparations are being made for the reception of 3,000 refugees at the Alexandra Palace, and the men ordinarily employed at the Palace have been taking the preliminary steps of scrubbing and laying down bedding for their reception. It is hoped to use the Alexandra Palace as a centre from which they can be drafted, as the arrangements of local authorities warrant, to suitable camps, or buildings for that matter, in the country.

### THE CANADIAN MILITARY HOSPITAL.

Sir William Osler, Regius Professor of Medicine at Oxford, and Mr. Donald Armour, both of whom are of course Canadians, have undertaken to organise and equip, for the Canadian War Contingent Association, a hospital of fifty beds for the use of the troops, to be known as the Queen's Canadian Military Hospital. The Association, of which the Hon. G. H. Perley is president, has arranged to equip and maintain the hospital for twelve months, and has power to raise the number of beds to a hundred should occasion require. The hospital, which will be under the control of the War Office, will cost for equipment and maintenance a sum estimated at £20,000, which, it will be remembered, is an amount similar to that decided on for the Welsh War Hospital, which was described in our columns last week. The Association also requires funds for its general work, and all with any inquiries to make should apply to Mr. J. G. Colmer, C.M.G., at the Westminster Palace Hotel, Victoria Street. It is possible that the building will be in London.

### ORGANISATION OF THE SCOTTISH RED CROSS.

So much has naturally been heard of Red Cross work in England that it is time a few points connected with the work of the Scottish Branch of the Red Cross Society should also be given prominence. Briefly, Scotland has been divided into four areas—Eastern, Central Eastern, North-Eastern, and Western—each of which possesses its Red Cross Commissioner, who represents the executive in his district, and is able to assist the counties in their work. The North-Eastern District, for example, comprises some half-dozen counties, and also Orkney and Shetland, with its central depôt at Aberdeen. The general scheme of work is, of course, familiar. The naval and military authorities make known certain requirements for the care of the sick and wounded in addition to those secured by the two Services, and the executive of the Scottish Branch then directs the attention of the counties to the matter. County lists are published of gifts received, which are stored and filed at the central depôt of the district. As to hospital accommodation, it has been stated that a demand for hospital accommodation in the North of Scotland is unlikely, since, as events have shown, the men for the most part have been transferred to the naval base hospitals in the south. It is therefore thought that the beds already available will be sufficient to cope with any cases that may arrive after sporadic engagements, and in this area, therefore, improvised hospitals are not being prepared till official instructions have been received. The same rule for the northern area also holds good as regards convalescent homes, though a list of suitable offers has been prepared. The recent landing of naval sick at Aberdeen, which we recorded, was an example of how, in this district at any rate, the already available beds have proved equal to the demands so far made upon them.

### NEW ADDRESS OF UXBRIDGE COTTAGE HOSPITAL

In its new home in Harefield Road the Uxbridge Cottage Hospital is now installed, and thanks to members of the Cox family the new site, Littleton House, possesses a full share of that quietness, air, and light which makes Denham, close by, so rare and delightful a bit of country under the very shadow of London. It will be observed, however, that the hospital is still in an adapted building, which is divided up in the following way. The ground floor accommodates the committee room, the kitchen, and the operating room; on the first floor are three wards, the women's ward being the largest with five out of the total of ten beds. The men's ward has four beds, and there is an isolation, or at least smaller, room with one bed. Thus the number of beds is an increase of three on that in the old Park Road building, and though for this and other reasons the change marks an aspiration. But even though a small institution, its well-wishers must work for the time when all adaptations will be discarded in favour of an attainment of the modern hospital standard which is only completely possible in a building specially designed for hospital purposes.

### WOMEN AND THE SCARCITY OF RESIDENT OFFICERS.

Last week we published a leading article on the subject of committees and the scarcity of resident medical officers, containing the suggestion that the difficulty "may have to be met by the introduction of such means as the appointment of supernumerary physicians and surgeons from practitioners resident in the area of the hospitals." On that suggestion we invite our readers' opinions. A letter appears on page 682 this week. In the meantime an analogous complaint has come from Miss Louie M. Brooks, secretary and warden of the London School of Medicine for Women. She remarks that there was a serious shortage of qualified practitioners before the war owing to the increasing demand for medical treatment and inspection, the competition of which has been one of the causes tending to deplete the supply of resident medical officers. She argues that owing to the war the entry at the medical schools for men will be much smaller than usual, and that "in five or six years' time there will, therefore, be a serious lack of house physicians and house surgeons." The only chance which she sees of remedying this is for many more women to enter on a full medical course this year. As an inducement to them to do so Miss Brooks states that she is receiving daily more applications from hospitals for fully qualified medical women than she can supply. The relation of the present need and opportunity for medical women here pointed out to the general scarcity of residents is obvious; but the new posts which are helping to absorb newly qualified practitioners also influence women doctors, and it is doubtful if a great increase of women residents is likely to be seen in five years' time. Still, if a large increase of women at the medical schools this year actually occurred, that would certainly be one of the possible prospects, although we are afraid that the solution of the difficulty will lie in rather different fields.

### THE PATRIOTISM OF THE BRISTOL GENERAL HOSPITAL.

At the half-yearly meeting of the governors of the Bristol General Hospital, which was held on Monday last, some remarkable details were given of the institution's policy in connection with the war. At the outbreak of hostilities the Royal Infirmary wrote asking whether, in view of their pledge to the War Office to place that institution at the disposal of the Government, the General Hospital could give help in any way by taking the Royal Infirmary's civil patients. The General Hospital replied that they would be willing to receive fifty surgical patients, but since then the Royal Infirmary has made such arrangements that the General Hospital's beds will not be required. The General Hospital has, therefore, patriotically decided, as best serving the community, to devote its beds for the use of the civil population, though, in proposing to open immediately the maternity ward, it will give preference to patients who are the wives of those serving in the Navy or Army. The hospital at the present time has a larger number of patients than usual, yet, nevertheless, five of the honorary physicians and three of the honorary surgeons are doing duty at the Royal Infirmary. Three of the honorary staff and the pathologist are also at the front. Should a need for additional military accommodation in Bristol arise, however, the hospital would be ready to receive military patients. We make a point of noting this attitude of the General Hospital, because it shows first that if one institution in a city offers, and is called upon, to devote all its accommodation to the Services in time of war, the other local hospitals may always be liable to become responsible for its work; and secondly, that Bristol General Hospital supplies a good instance of the equal patriotism, under proper circumstances, of a hospital in war-time deciding to devote its whole accommodation to its first claimants, the civil population, alone.

### THE WEEK'S DRUG MARKET.

Nothing has occurred as yet to relax the stringency in the Drug market consequent upon the shortage of supplies brought about by the war. In a few cases quotations have continued to advance, but, generally speaking, the market shows a tendency to settle down to the new conditions, and while business is naturally slow and restricted to small transactions, the excitement which was noticeable immediately after the outbreak of hostilities has abated. The only advice it is possible to give to buyers is to secure, if possible, such quantities of stocks as are needed for immediate requirements; indeed, this appears to be the only course open. Some supplies of potassium bromide have arrived from America, and this fact should tend to check further advances in price; as dispensers are aware, however, American bromides are not esteemed quite so highly as bromides of European production, but under existing circumstances the American product will be welcomed. An auction of senna was held in London last week, and the supplies offered sold at a substantial advance. It is satisfactory to note that a number of applications have been made by British firms for permission to work patents for chemical products used in medicines, of which the owners are subjects of States at war with His Majesty. In view of the fact that we are largely dependent upon various parts of the European continent for medicinal plants, it goes without saying that the prices of most of these have advanced considerably. As one instance chamomiles may be mentioned; this plant is grown in large quantities in or near districts which have been the scenes of much of the fighting in Belgium; the crops were ready just about the time when war was declared, and it is obvious that they cannot have been harvested under normal conditions.

